# Perforated Appendicitis in a 52-Year-Old Male With Previously Undiagnosed Intestinal Malrotation: A Case Report

**DOI:** 10.7759/cureus.106530

**Published:** 2026-04-06

**Authors:** Afnan A Alotaibi, Masoud A Alghamdi, Qussay R Wazzan, Abdulrahman A Alzahrani, Ahad T Banjar

**Affiliations:** 1 General Surgery, King Fahad General Hospital, Jeddah, SAU; 2 General Surgery, King Fahad Armed Forces Hospital, Jeddah, SAU

**Keywords:** adult, computed tomography, intestinal malrotation, left-sided appendicitis, perforated appendicitis

## Abstract

Intestinal malrotation is a congenital anomaly resulting from abnormal midgut rotation during embryologic development. Although commonly diagnosed in infancy, it may remain asymptomatic until adulthood and is often discovered incidentally. Acute appendicitis in the setting of malrotation may present atypically due to abnormal positioning of the cecum and appendix, potentially leading to delayed diagnosis and increased morbidity.

We report a case of a 52-year-old male who presented with left lower quadrant (LLQ) abdominal pain and was found to have perforated appendicitis in the context of previously undiagnosed intestinal malrotation. Computed tomography demonstrated right-sided small bowel, left-sided colon, reversal of the superior mesenteric vessels, and a displaced, inflamed appendix with associated abscess formation. Surgical exploration confirmed malrotation and perforated appendicitis, requiring conversion from laparoscopic to open appendectomy. The postoperative course was uneventful.

This case highlights the importance of considering intestinal malrotation in adults presenting with atypical abdominal pain suggestive of appendicitis and emphasizes the critical role of computed tomography in diagnosis and operative planning.

## Introduction

Intestinal malrotation is a congenital anomaly caused by incomplete or abnormal 270° counterclockwise rotation of the midgut around the superior mesenteric vessels during fetal development [1,2]. The estimated incidence is approximately one in 200-500 live births, with symptomatic cases occurring in nearly one in 6,000 neonates [1,2].

Although malrotation commonly presents in infancy with volvulus or obstruction, adult cases are rare and often asymptomatic [2,3]. When symptoms occur in adults, they are typically nonspecific and may lead to delayed or incidental diagnosis. Acute appendicitis, one of the most common surgical emergencies, may present atypically in the presence of malrotation due to abnormal positioning of the cecum and appendix [4,5].

We present a case of perforated appendicitis in a middle-aged adult with previously undiagnosed intestinal malrotation.

## Case presentation

A 52-year-old male with a history of type 2 diabetes mellitus managed with oral hypoglycemic agents presented with a two-day history of progressive abdominal pain. The pain initially began in the periumbilical region and subsequently localized to the left lower quadrant (LLQ). He denied nausea, vomiting, or changes in bowel habits but reported subjective fever.

**Figure 1:**
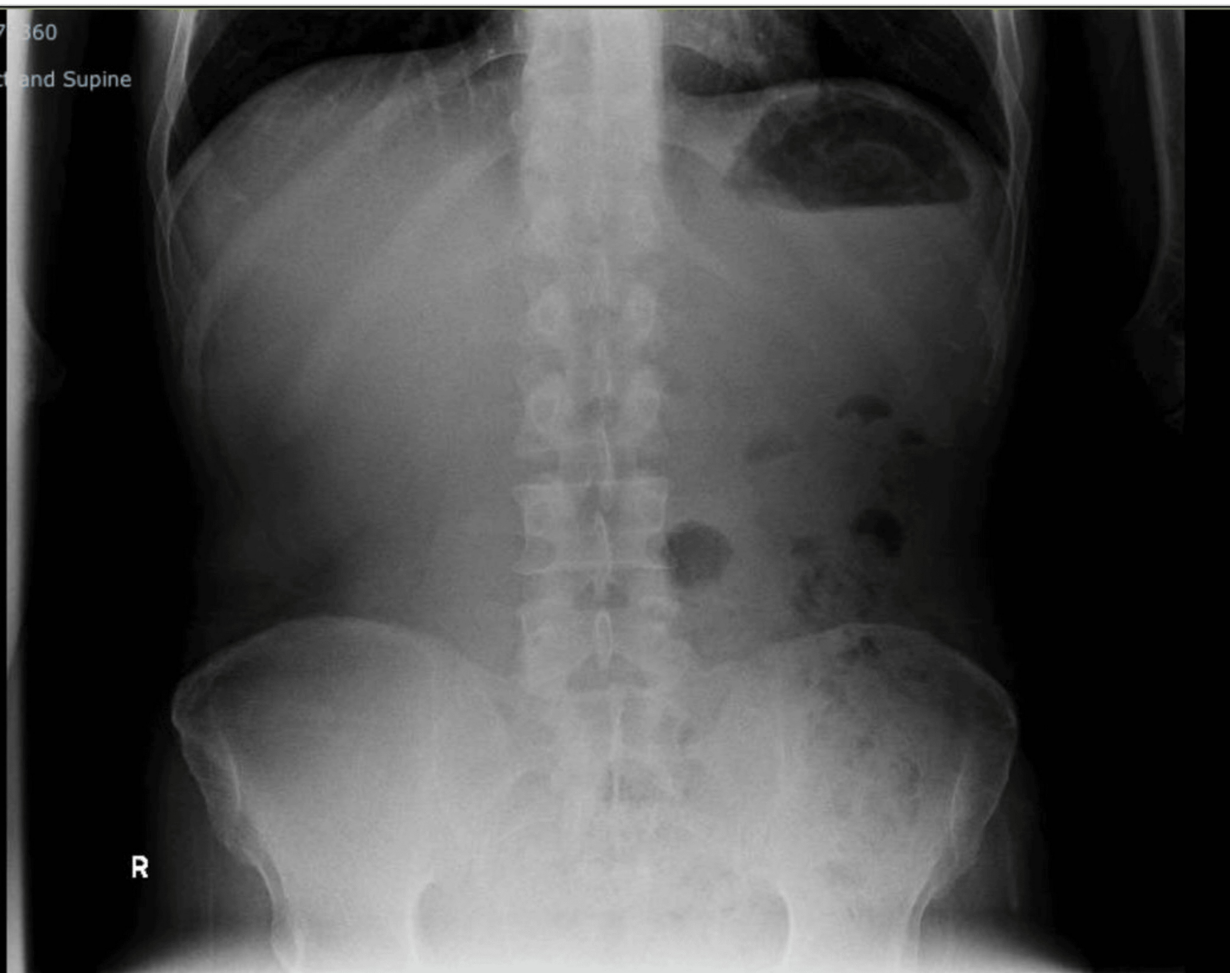
Plain abdominal radiograph Anteroposterior view demonstrating a nonspecific bowel gas pattern without evidence of obstruction.

On examination, the patient was afebrile (36.5°C) and hemodynamically stable (heart rate 91 bpm, blood pressure 132/89 mmHg, respiratory rate 18 breaths per minute, oxygen saturation 99% on room air). Abdominal examination revealed LLQ tenderness with positive Rovsing’s and Dunphy’s signs, without signs of generalized peritonitis. Laboratory investigations demonstrated leukocytosis (white blood cell count 12 × 10⁹/L). Plain abdominal radiography was unremarkable (Figure 1).

**Figure 2:**
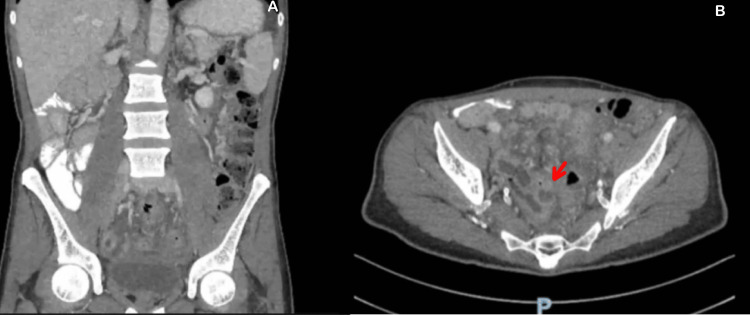
Contrast-enhanced CT scan Demonstrating (A) right-sided small bowel and left-sided colon consistent with intestinal malrotation, and (B) peripherally enhancing tubular structure (arrow) representing an inflamed appendix with associated pelvic abscess.

Given the atypical localization of pain, contrast-enhanced abdominal CT was performed. CT imaging demonstrated small bowel loops predominantly on the right side and colon predominantly on the left side, with a reversal of the superior mesenteric artery (SMA) and superior mesenteric vein (SMV) relationship, consistent with intestinal malrotation [3,6]. The appendix was not visualized in the right lower quadrant. Instead, an irregular, peripherally enhancing tubular structure measuring approximately 1.5 cm was identified near the displaced cecum. A fluid collection measuring 3.6 × 5.8 × 5.3 cm containing internal calcified foci was noted in the rectovesical pouch, suggestive of perforated appendicitis with abscess formation (Figure 2).

Laparoscopic exploration was initiated with anticipation of a left-sided appendix. Due to complex anatomy and limited visualization, the procedure was converted to an open approach. Intraoperatively, malrotation was confirmed. Adhesiolysis and cecal mobilization were performed, and the appendix was identified in the retrorectal cul-de-sac. A transfixation appendectomy was completed, and the operative field was irrigated.

The postoperative course was uneventful. Histopathological examination confirmed perforated appendicitis. The patient tolerated oral intake and was discharged on postoperative day four in stable condition.

## Discussion

Intestinal malrotation results from abnormal rotation and fixation of the midgut during embryogenesis. While it frequently presents in infancy, adult presentations are rare and heterogeneous [2,6]. Many adult cases are discovered incidentally during imaging or surgery for unrelated conditions [5,7].

In normal anatomy, acute appendicitis classically presents with periumbilical pain migrating to the right lower quadrant. However, in malrotation, the cecum and appendix may be located in atypical positions such as the LLQ, midline, or pelvis. This altered anatomy may mimic diverticulitis or other intra-abdominal pathology, contributing to diagnostic delay [5,8]. CT imaging plays a crucial role in diagnosing both malrotation and complicated appendicitis. Characteristic findings include right-sided small bowel, left-sided colon, and abnormal SMA/SMV orientation [3,6]. Recognition of these findings is essential to guide surgical planning and prevent intraoperative surprise.

Left-sided appendicitis associated with malrotation is uncommon but documented in the literature. A review by Akbulut et al. analyzing 95 cases highlighted frequent diagnostic delay due to atypical pain localization [8]. Similarly, Hou et al. emphasized the diagnostic value of CT in identifying aberrant appendix positioning [9]. Failure to recognize malrotation preoperatively may complicate surgical management. Although Ladd’s procedure is recommended in symptomatic malrotation to prevent volvulus, management in asymptomatic adults remains controversial and should be individualized based on clinical context [7].

## Conclusions

Intestinal malrotation should be considered in adults presenting with atypical abdominal pain suggestive of appendicitis. Early CT imaging is essential for accurate diagnosis and operative planning. Awareness of abnormal bowel positioning can prevent diagnostic delay and reduce complications such as perforation and abscess formation. Prompt surgical intervention results in favorable outcomes.
